# Design Thinking as a Means of Citizen Science for Social Innovation

**DOI:** 10.3389/fsoc.2021.629808

**Published:** 2021-05-07

**Authors:** Hoe Chin Goi, Wee-Liang Tan

**Affiliations:** ^1^Graduate School of Management, Nagoya University of Commerce and Business (NUCB) Business School, Aichi, Japan; ^2^Lee Kong Chian School of Management, Singapore Management University, Singapore, Singapore

**Keywords:** citizen science, design thinking, social innovation, citizen persona, citizen intermediary

## Abstract

Members of the public or community can play a significant role in the development of social innovations. When a social innovation is developed involving a scientific approach and the community, there is the confluence of two fields-citizen science and social innovation. Social innovations can be developed through the employment of design-thinking. In this paper, we advocate design thinking as an approach to marry the two fields for a desired outcome of improved community life in ageing housing estates in Tokyo. The two fields, citizen science and social innovation, are described in brief before the design thinking method is introduced and its utility in engaging citizens for social innovation is explored. The paper provides two case illustrations and the lessons drawn from them. We conclude with pointers for others who desire to employ this approach. When the resultant innovation and design-thinking approach are adopted by the community for future projects, there could be a change in society and possible forward movement for self-help and change.

## Introduction

Citizen science has made relevant the work of scientists as it involves sections of the community and public in the activity of science (Irwin, 1995). Citizens have been engaged in a variety of scientific projects; for example in ornithology (Bonney, 1996), or scientific work undertaken by members of the general public, often in collaboration with or under the direction of professional scientists and scientific institutions (European Commission, 2016, p. 54). With such engagement, members of the public become aware about science. Their involvement promotes greater interest and a potential increase in scientific activities. There is an increasing number of projects where citizens participate in contributions typically in the area of data collection. Engaging the citizens in the measurement of phenomena benefits both science and citizens through the discoveries, applications, and policy decisions that the participation of citizens enable.

In addition to the awareness and engagement of the citizens, the citizens benefit from the scientific findings and results by the increase of knowledge or practical consequences that result from the science. One other area citizen science can benefit citizens in a more immediate and direct manner, is when the scientific efforts led to solutions in the form of social innovations, addressing social problems that affect groups of people or communities. Social innovations could be developed by individuals (social entrepreneurs), organizations, or even the community. The development process could involve science or scientific approaches. The science might not be at the level of discovery but at the level of human sciences because in many solutions, there is a need to understand needs and ascertain suitable approaches to address the social problem. It is this intersection of science, citizens, and social innovations, that this paper addresses.

In this paper, it is suggested that citizen science can extend into the realm of social innovation with the citizens involved in citizen science through the design thinking approach. There are two settings where citizen science can take place in the development of social innovations. A scientist (social entrepreneur) could initiate a project which involves the citizens in data collection with the solution that the project envisages being intended for them as subjects of the science. For example, an agriculturist could conceive of a way in which new forms of soil improvement could be introduced in a region. Such a project could involve soil measurements, sample collection, understanding of the farming methods, or sources of irrigation among other things. The community could be enlisted to participate in data collection and to provide the necessary information. At other times, the community could be the scientists in applying science themselves to resolve problems they encounter. The first setting is employed in this paper to explore the role citizen science could play in social innovations. Two projects have been initiated by the lead researcher with two communities where the residents have participated. Accounts of these two projects are presented to illustrate the manner in which citizen science have been employed in developing social innovations for these two communities with design thinking as the methodology.

The paper is divided into five sections. After this introduction, the paper examines the potential for the development of social innovations through citizen science with design thinking as the approach employed. The authors provide two case illustrations in section Projects. The two projects tell of how residents in suburban towns in Japan (citizens) participated in university-led projects by employing design thinking to develop prototypes (social innovations). Section “Contributions of Citizen Science to Social Innovation” describes how citizen science contributes to social innovation through design thinking. In the concluding section, the authors suggest recommendations for the employment design thinking for citizen science.

## Citizen Science, Social Innovations, and Design Thinking: Additional Opportunities

### Citizen Science

The involvement of scientist-supervised citizens (laypeople or volunteers) in research with the use of the citizen science tools have been a boom for scientific publications with exponential growth of citizen science publications in indexed journals for the last two decades (Sanz et al., 2021). Citizen science has benefited science and mankind through the projects that have proliferated despite the lack of an agreed definition of citizen science. Eitzel et al. (2017) provided an account of the various terms employed in citizen science across different fields, geographical contexts, that has led to consternation on the part of some scientists who prefer standardization (e.g., Heigl and Dörler, 2017).

Mahr et al. (2018) highlighted the co-production of reflexiveness and dialogue between citizen science practitioners and researchers in a bilateral relationship. Danielsen et al. (2018) focused on inclusion of indigenous and local knowledge in citizen science for innovation from a bottom up perspective. Wyler and Haklay discussed the potential of citizen science to be integrated into university research, but have fallen short to taking reference in methodological approach, such as design thinking. In education, citizen science has been utilized to engage the public via storytelling and visualization techniques (Sforzi et al., 2018). Novak et al. (2018) discussed different forms of citizen engagement with a participation model to create participatory digital social innovation.

### Social Innovation

Social innovation is a concept McGowan et al. (2017) traced to a sociologist, Ward (1903). It was employed by an economist, Schumacher (1973) to highlight intermediate technology to solve the social and economic problems of the poor. In more recent times, interest in social innovation arose with the quest for solutions to address social problems. With the myriad social problems, it was realized by stakeholders such as policymakers that creative solutions were needed. Governments and policymakers are often far from the social problems on the ground. Solutions were needed for these wicked problems (Rittel and Webber, 1973); solutions that fit their contexts. More often than not, the development of the solutions called for the mobilization of people. The efforts are not limited to the policy makers or philanthropists. They include social entrepreneurs, bureaucrats, frontline staff, service users, observers, or volunteers (Mulgan, 2007). Social innovations include technology and also frameworks of insurance and healthcare which have a huge impact on society (Drucker, 1985). Efforts in developing social innovation entailed the generation and implementation of new ideas, and the organization of interpersonal activities or social interactions to meet one or more common goals. They could also involve providers of products and services (Von Hippel, 2005) or result from consumer-company interactions (Prahalad and Ramaswamy, 2004).

Of interest to this paper is the involvement of users and the disadvantaged whom the solutions are to benefit. Urama and Acheampong (2013), for instance, report the engagement of the slum dwellers in Kenya. Matsushima and Takahashi (2007) included users and environments, explaining in their article about how social innovation often required a new perspective to clarify the dynamic process in which institutional entrepreneurs come to co-opt and make relational rules with various actors. Similarly, Tanimoto (2012) clarified the emergent process of creating social innovation in collaboration with stakeholders in the local community. Social innovations were viewed as a subset of innovation as were inclusive innovations and grassroots innovations (Tan and Zuckermann, 2019). However, the prior research did not further explore the specific roles and effects citizens could play in social innovation. While they examined the role of communities in social innovation, they did not conceive of them as citizens or citizen scientists. Herein is an overlooked intersection that warrants attention.

### Citizen Science, Social Innovation, and Design Thinking

It stands to reason from the foregoing that citizen science can be employed in the development of social innovations. Eitzel et al. (2017) noted that terms describing citizens include “anonymous, non-identified,” “amateur, hobbyist,” “citizen,” and “citizen/individual citizen scientist.” It is telling that they observed that “citizen” was defined as “an inhabitant of a particular town or city; a member of the general public in a defined geographic locale.” Hence, when citizens who are members of a community could be engaged in citizen science as “citizens.” As to the scientists in citizen science, they noted that the terms employed include “citizen scientist, scientist-citizen, public scientist, community scientist” defined as “individual with formal science training who is actively engaged in the civic sphere and wants their work to both serve the greater good and do so transparently.” Hence, a scientist seeking to develop a social innovation could qualify to be engaged in citizen science if members of a community or town participate in providing the information required.

The common ground between citizen science and social innovation is the role of citizens, and more so, in the case of citizen science by definition than in social innovation. A solution could be adapted from one country for introduction in another with the community, beneficiaries, or users coming on board in the phase of implementation. Citizen science speaks of their involvement in science at an earlier phase in the development phase. One bridge that links citizen science with social innovation is the scientific approach employed. In this paper, design thinking is suggested as this bridge. Design thinking is an approach or method in the same mould as another research method a citizen scientist might employ. It has a number of advantages.

Design thinking is a scientific approach to innovation that is human-centered. Design thinking has been applied to resolve social problems and create solutions as applied in many fields. For example, students from Stanford school employed design thinking to help developing regions to create solutions as social innovation projects (Brown and Wyatt, 2010). The method has the characteristics of being user centric, process oriented due to its ethnographic richness in deriving empathy of end users through face-to-face observation and direct interview. The steps in design thinking, thus, include research methodologies common to science. To obtain user inputs, there may be interviews, participant observation, focus groups, or surveys. The engagement with the citizens is evident in scientific methods employed.

Popularized by its use in industrial design by IDEO (Brown, 2008), it draws from the designer's toolkit to integrate the needs of people, the possibilities of technology, and their requirements for business success (Brown, 2009). Design thinking research has been discussed in different fields including design (Simon, 1969; Schön, 1983; Buchanan, 1992; Cross, 2006) and management (Martin, 2009). Design has been one area where citizens have been engaged in co-development.

Citizen science can employ design thinking as an approach in social innovation projects. It offers certain benefits. First, design thinking places emphasis on the role of users, the citizens, in projects. Second, it could be employed without using large sample sizes as many design thinking projects involve the target user as a representative of the whole. Third, the community is able to see the output from the projects in the form of prototypes which could take the form of simulations, programme designs, webpages, or mock-ups. Fourth, there could be the adoption of the recommendations for implementation by the community. This is a key reason the authors suggest it as a method that could be used by the various stakeholders to engage citizens in science as the outcomes/solutions can be reviewed by the community for implementation. Fifth, the visibility of citizens, who are peers from the community, implies endorsement by members of the community and there is potential of identification of the other citizens, who are not involved in the projects, with the project or research being conducted.

In the following section, we describe two projects involving citizens in design thinking for the development of social innovations.

## Projects

### Overview of Projects

In these two projects, the scientist is the first author, who with his student teams worked with citizen scientists, the residents of the two communities, using design thinking to develop solutions to address a social issue faced by both communities: ageing seniors and the need for active living on their part as they age in place.

The projects involve collaboration amongst residents, representatives from the local communities (housing estates one each from two suburban Japanese regions, Kimi No Mori town in Chiba prefecture and Ena city in Gifu prefecture), university student teams, and other external stakeholders. The university student teams comprised international students from Nagoya University of Business and Commerce (NUCB).

### Context of Projects

The rapidly ageing population in Japan requires innovative solutions for seniors to lead active lives where they are in their communities. Social innovations are much accepted in Japanese society. They are often driven by formal Japanese corporations such as OMRON Taiyo Home Co., Ltd. with initiative coming from the corporations (Fujisawa et al., 2015), or by social enterprise, such as Hokkaido Green Fund (HGF) for the development of energy business from stakeholders' perspective (Tanimoto, 2012).

The sites for the projects are typical of ageing suburban regions in Japan. These suburbs typically have the ageing towns with typical “fading” signs. Most residents are able-bodied, financially independent, and skillful retirees, who reside alongside some pockets of young families. The towns have relatively good infrastructure but are less accessible to big cities, such as Tokyo or Nagoya, respectively. These suburban regions encompass nature reserves and possess unique cultures.

Kimi No Mori town is about 50 kilometres away from Tokyo and accessible by train or highway. The estate was developed by Tokyu Land Corporation in the 1980s. The uniqueness of each Tokyo Land Corporation estate is the development of a residential area within a golf course, with customized building architecture resembling an American district. Thus, the estate appealed to residents who enjoy exclusive countryside residential and the “overseas” experience. Kimi No Mori has close to 3,754 residents of about 1,485 families. Those aged above 65 years old make up about 24% (899 people). Out of this group (65 and above), 72% were permanent residents, while the rest of 28% are residents but have the intention of leaving. Within the town, there is a group of retired residents who promote an “organic lifestyle and good dietary habits.” This group cultivates natural products, such as organic vegetables, organic blueberry, and produces small scale commercial items for sale within the town.

Ena city is larger with about 50,000 residents. It is about 70 kilometres away and about an hour car ride from Nagoya, the most bustling area in Central Japan. One of the unique features of Ena city is that it lies along “Nakasendo” highway, one of the two key routes from Tokyo to Kyoto in the Edo period in the 1800s. It is known as the “49th station” along the Nakasendo highway, where the Emperor has stopped over to stay. There are many facets in Ena's cultural life today - several festivals, activities, art creation, and local products - to mark significant historical events. The “noren” split curtain festival is one such event held annually around October till December. A “noren” is a split curtain that is culturally hung on the door or local restaurants, shops, or even houses as a decoration and identification of the business owner's craft. During the festival, about 200 noren curtains will be hung along the streets in Ena city, as a way of celebration and depict the vibrancy of the city in the Edo period.

Both project sites were selected by the researchers as the “context” for social innovation employing design thinking to explore innovation of new solutions. Within the two projects, four citizens from the local communities participated in the projects as citizen scientists. The roles are described below in section The Role of the Citizens in the Research and summarized in Table 1.

**Table 1:** Samples of citizen science projects.

**Case**	**Case study I**	**Case study II**
Citizen typology	Project in Kimi No Mori town (2018)	Project in Ena City (2019)
Citizen Persona (CP)and role in Design Thinking	Ms Kitakaze Resident and Organic Blueberry Farmer Provide organic blueberry jam tasting experience for student designers Sharing of experience and needs through social media Feedback on final prototype and proposal	Mr Kato Resident and citizen committee head for noren split curtain festival Conduct experiential learning of mini noren contest in class for students Sharing of experience and needs in class Feedback on final prototype and proposal
Citizen Intermediary (CI) and role in Design Thinking	Mr Yuki Hara Committee member in the residents' club Interpret, iterate and evaluate prototype and proposal throughout Design Thinking stages	Ms Naruse Ai Resident and staff of International Exchange Association in Ena city Interpret, iterate and evaluate prototype and proposal throughout Design Thinking stages

### Employment of Design Thinking With Citizen Science in Two Communities

#### Implementation of Design Thinking

The projects were conducted as part of an undergraduate course on design thinking for international students from two cohorts in 2018 and 2019, respectively. The coursework requirement for the course is the development of social innovations to promote active living in the two estates. In phase one of the course, the researchers introduced the student teams to social innovation and design thinking within the classroom environment through case studies. They are instructed on six steps in the design thinking process: “empathy,” “definition,” “ideation,” “prototype,” “iteration,” and “testing.” The student teams were then introduced to the contexts they were to apply design thinking through a series of speakers: the researchers, representatives from the two estates and Tokyo Land Corporation representatives. They presented the facts, problems faced in each estate, current activities, and future goals in the estates.

The second phase involved the students engaging with the citizens in design thinking. With the lectures and speakers, the student teams were exposed to the contexts of the estates and able to empathize with their situation. Through empathy, they were able to identify the needs in the estates before entering the creative step where they think of solution-ideas. They developed the prototypes of the solutions which they would iterate with a few residents before the final evaluation in the form of a presentation before a panel of residents and stakeholders. Local citizens were invited to participate in these three stages, “empathy,” “prototype,” and “testing” via face-to-face or online connection format. The resident representatives either came to class or participated over SKYPE meetings^1^ with ideas from the student teams being presented when they shared their computer screens with the citizens in the SKYPE meeting. The students interviewed and conducted surveys on the citizens as samples of the community at the “iteration” stage.

Those sessions of feedback enabled students to obtain direct feedback from residents and stakeholders to improve the idea and prototype. A post mortem session was carried out between the researcher and the residents directly at the end. The phases and activities are shown in Table 2.

**Table 2:** Timeline for employing design thinking in two suburban towns.

**Cohort/year**	**Month**	**Activity**	**Action**
One in 2018	Feb	Planning for Action Research	Formalization of joint research
	Mar (Phase 1)	Design of content for project for Kimi No Mori Estate”	Formalization of content and system
	April - July (Phase 2)	Implementation of “Design Thinking course-Kimi No Mori Estate”	5 solutions and prototypes were created
		Presentation of outcome to residents	Resident kept the proposal for consideration
		Post mortem lesson discussionShowcase of “Digital Blueberry” video at film festival in university	Feedback Won award for “Community Film” category
Two in 2019	Aug (Phase 1)	Design of content and system project for Ena estate	Formalization of content and system
	Sept- Dec (Phase 2)	Implementation of “Design Thinking course- Ena Estate”	5 solutions and prototypes were created
		Presentation of outcome to Ena residents	Residents were impressed with the proposal. There is strong interest to pursue the proposal. Invitation of students to implement in town
		Presentation of Prototype and solution at noren festival opening ceremony	Attended by Ena city mayor, government officials and residents

#### The Role of the Citizens in the Research

Two citizens from each city are invited as the engaged citizens. They have volunteered to be part of the design thinking project to support the creation of social innovation that would impact their local community.

The citizens play the roles of citizen persona, the target user of the solution to be designed, and as citizen intermediaries, liaising between the scientists (researchers and student teams), and the citizens. Citizen persona are individual residents from the respective estates who provide information about their situations, needs, and issues faced. In the design thinking approach, “persona” would be a representative on behalf of a population of end users to reflect the background, latent needs for empathy by designers. In addition, citizen persona will access and provide local resources which are prescribed as “raw materials” relevant for the solution design.

In Kimi No Mori estate, Ms Kitakaze, in her sixties, a long-time resident, who retired after a successful career as designer for Tokyo Disneyland, is the citizen persona. In her retirement, she cultivates and produces organic blueberries in her own garden with her family. They shared the motivation to create unique organic blueberry jam which is good for vision health, especially for young working adults who strain their eyes working on computers, as well as seniors who have declining eyesight due to ageing. She is entrepreneurial, motivated not to profit, but to create something by using her curiosity to contribute towards her community. In spite of her elder age, she enjoyed posting her activities on social media, such as Facebook and Line. Her motivation for working on the project is to bring more people to her town in the light of the dwindling of activities in Kimi No Mori estate.

In Ena estate, Mr Kato, in his seventies, a male local resident leader of the annual “noren” festival, is the citizen persona. His committee receives fullest support from the city mayor, local merchant association and local schools in Ena city. He is very motivated and receptive to making incremental changes within his means that contribute towards his community. He is not technologically savvy with social media but loved to find opportunities to connect with others. Even though he had past experiences teaching students to make “noren,” he has not collaborated with any university on any formal research activities.

Citizen intermediaries are either residents or individuals with connections to the communities in the estates. They act as liaison amongst the scientists (researchers and student teams), the citizen persona and the community at large. In addition, citizen intermediaries will interpret any tacit knowledge, such as experiences and emotions of the residents. They play an important role in the iteration of prototype A citizen representative from each estate is invited as citizen intermediaries into the projects. They are motivated to be the “middleman” to share the narratives and to bring social impact to their respective community.

Mr Yuki Hara, about 30s, Japanese, a committee member in the residents club in Kimi No Mori estate. He does not physically stay in the estate, however has been an active volunteer who regularly homestay and visit the estate due to his close ties with the residents. He has committed to research and implement solutions that promote local participation amongst residents and other surrounding stakeholders, such as schools, universities, and industrial organizations.

Ms Naruse Ai, about 40s, Japanese lady works as a government representative in the International Relation Department in Ena city, within Gifu prefecture. She has joined the organization for about a year and is currently responsible to promote international collaboration between Ena residents and the foreigner residents in Ena city, as well as any external international partners or communities.

#### Student Teams

The student teams comprised two cohorts of international students. There were 27 students from the first cohort in 2018 and 24 students from the second cohort in 2019. In each cohort, five student teams were formed. The students were mainly business background, undergraduates, however with a few master level and engineering backgrounds. They were mostly not able to speak Japanese and were unfamiliar with the Japanese local communities. They were tasked to develop solutions that promote active living by the elderly communities in suburban areas as their course project. Thus, the students became actors for citizens to effectuate and collaborate through design thinking within the citizen science framework.

### Solutions Suggested for the Estates

#### Solution Selection

The student teams developed a number of solutions per estate. From the developed solutions, two solutions with the highest scores when evaluated by the citizen persona and citizen intermediaries were selected. The selection criteria were the degree of engagement between the residents and the student teams, solution innovativeness, solution implementation feasibility, and the degree of desirability. The residents and stakeholders as beneficiaries rated the solutions on their desirability. The selected solutions are namely “Digital Blueberry” video by Team A from the first cohort for residents in Kimi No Mori estate, and “Harmony Audio” system by Team B from the second cohort for residents in Ena estate. The citizen engagement to create the solutions are summarized in Table 3.

**Table 3:** Process of citizen engagement to create solutions.

**Lesson**	**Key content**	**Design thinking coursefor Kimi No Mori Estate (2018)**	**Design thinking course for Ena estate (2019)**
1	Understanding design thinking	Context of Kimi No Mori estate by Citizen Intermediary (Mr Hara)	Context of Ena estate by Citizen Intermediary (Ms Naruse)
2	User's empathy	Empathy through Citizen Persona (Ms Kitakaze) (online) Tasting of Blueberry Jam	Empathy through Citizen Persona (Mr Kato) Experiential learning of “noren” curtain design in classroom
3	Define wicked problem	Team discussion	Team discussion
4	Ideation	Brainstorming	Brainstorming
5	Prototype marking	Prototype filming	Application making
6	Iteration	Iteration of prototype withMr Hara	Iteration of prototype with Ms Naruse
7	Testing (Final proposal presentation)	Evaluation and feedback by Mr Hara and Ms Kitakaze (online)	Evaluation and feedback by Citizen (Mr Kato and Ms Naruse) (Face to Face)

#### Solution I: “Digital Blueberry” Video for Kimi No Mori Estate

At the “empathy” stage, Team A, made up of five international students, tried to gain a deeper understanding of the residents' situation and empathize through secondary sources: the social media and past records about Kimi No Mori town, They were able to gain insights from Ms Kitakaze's video, photographs, and social media posts about her cultivation and sales of organic blueberries. Greater empathy for their idea resulted from tasting Ms Kitakaze's blueberry jam which she delivered to the team.

In the “definition” stage, Team A tested various ideas. They set out to produce a short advertisement which was both informational and captivating for locals and others who lacked a knowledge of Kimi No Mori by using the story of Ms Kitakaze san and her blueberries. They identified Kitakaze as the “Disneyland Lady” from her career as designer of Disney's costumes, in particular, the Mickey Mouse ones. They were inspired to use animation in their video as fit with the youthful and fun theme in Disney and it reflected her past experience and current mindset. An additional inspirational factor was the health benefits that the organic blueberry jam provided in improving vision health. The team found that blueberries was a good message to send as it drew attention to the elderly residents in Kimi No Mori town, and to the good quality organic jam produced there. This message could serve as a bridge for the town to youth groups attracting them to preserve the environment and to eat healthily.

At the “prototype” stage, they decided upon innovative content with a key characteristic. The content would compose a hybrid of both real-life footage and an animated blueberry icon, named “Jerry,” which would be included in a storytelling format. The intent was to make the video more youth-friendly and appealing to a wider audience.

During the “iteration” stage, Mr Hara, the citizen intermediary, contributed his feedback to the student team through online interaction during the class and subsequently by email. He highlighted that Japanese viewers would not be able keep up with the conversation in video without Japanese subtitles which he suggested be added to make the video understandable to non-English speaking audiences. Through the repetitive “trial and error” sessions, the prototype video was filmed and created using the “green screen technology” as taught within the course.

At the “testing” stage, Ms Kitakaze, Mr Hara, and a few external stakeholders, who formed the panel of evaluators, viewed the final version of the advertising “Blueberry Jam” video. They were connected using an online and synchronous communication platform, SKYPE in class. Both residents provided additional feedback on how to build social networks to physically purchase the blueberry jam if there was interest on the part of the audience.

Team A leader said “We were fortunate to communicate and received in-depth feedback through Mr Hara, based on the initial prototype and proposal of their project. This proved extremely useful, as it provided a rather unique take and view of the video pointing out areas of improvement we had not even considered.”

The “Digital Blueberry” video proposal was evaluated to be one of the most creative solutions. Ms Kitakaze was pleased to consider using the digital movie to market her blueberry jam. Other foreigners in the panel were also impressed and felt a sense of “relationship” with Ms Kitakaze and her product. They were also curious what made Kimi No Mori town such as an “unknown” town that brought “hope” and “activities” to the community.

**Figure 1:**
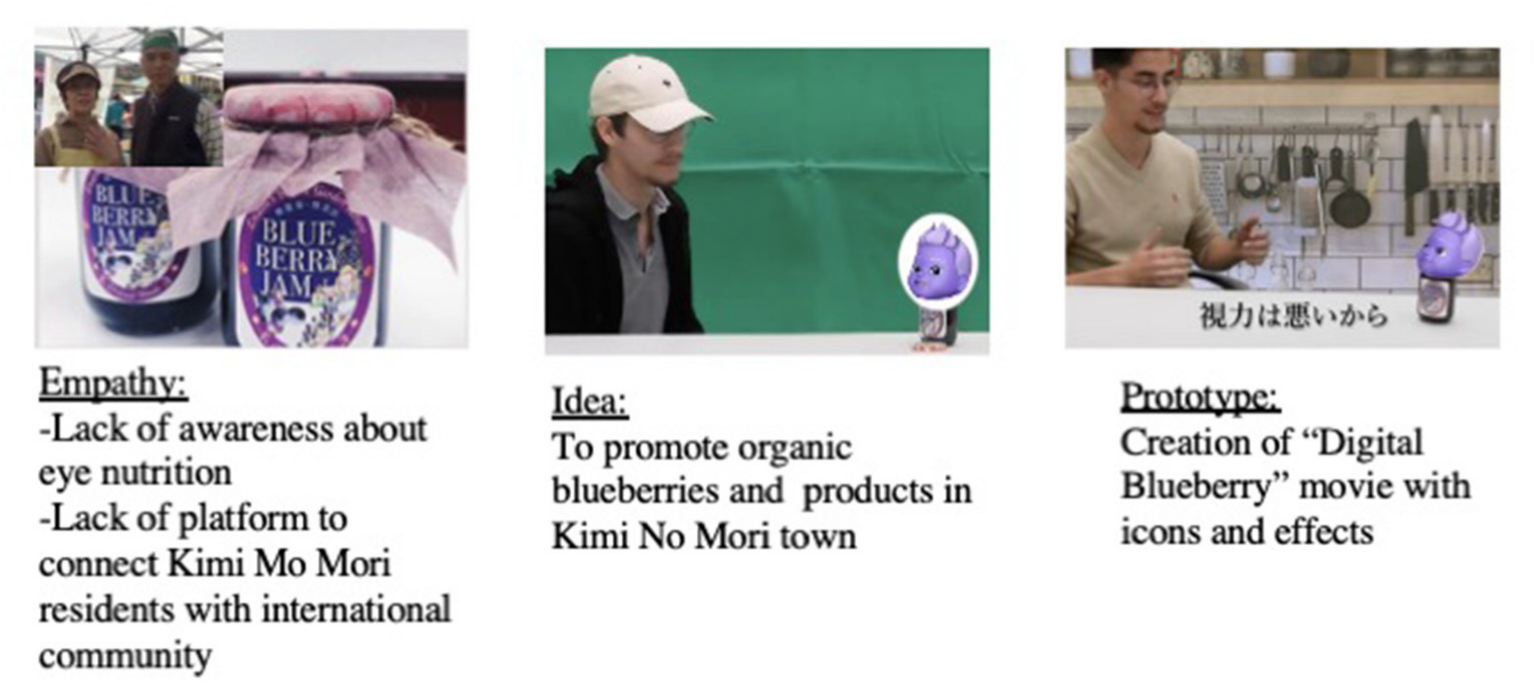
“Digital Blueberry” video for Kimi No Mori estate.

After the project, a few members presented their digital movie at the NUCB Film Festival to about 100 student audiences. The film won a prize under the “community film” category. The narratives and learnings of the project were also written into three separate case studies registered under NUCB case centre for educational purpose. The prototype and outcomes are summarized in Table 4 and Figure 1.

**Table 4:** Social innovation through design thinking.

**Projects**	**Items**	**“Digital Blueberry” video**	**“Harmony Audio” system**
Social innovators and prototype	Design team	Team A from first cohort in 2018 (5 members)	Team B from second cohort in 2019 (5 members)
	Background	Diverse nationality Undergraduate level	Diverse nationality Undergraduate level
	Prototype by team	Video that promote and make the organic blueberry jam and its origin from Kimi No Mori town	System that enable tourist to under attraction in Ena city via QR-Code system
Outcomes	Educational contribution	Case study registered with case centre in university	Case study registered with case centre in university
	Social innovation	Digital Blueberry VideoPresented at film festival and won the “community film” award in the University	Noren Design are used for Noren Festival Presented to Mayor and about 150 residents at launching ceremony of noren contest in Ena city

#### Solution II: “Harmony Audio” System for Ena Estate

Team B hoped to establish a shared economy in the Ena estate to promote the exchange of data and resources, develop peer to peer relationships, and create economic benefits.

The team received information about Ena-city through local brochures and publicity materials, which enabled them to visualize the town and its surroundings. To further empathize with the residents, Mr Kato gave an introductory lecture about Ena town and “noren” curtains as a Japanese culture. A mini “noren” design contest was organized for the entire cohort, including members in Team B. While making the noren curtain, the team took the opportunity to interview Mr Kato. Through the face-to-face interaction, students could gain additional insights about the needs of the Ena residents, the importance the resident attached to noren and began to identify with the culture.

At the “definition” stage, the team identified the social issues faced by residents in Ena estate. They realized there was a lack of awareness about the attractions in Ena city. The existing publicity materials were static marketing brochures and official websites in Japanese. There was insufficient being done to attract tourists and, as a consequence, lack of awareness about its attraction. Most foreign visitors either did not know about the existence of the city or they did not know what there was to do there in terms of attractions. Thus, Team B decided to focus on these problems. They defined the wicked problem as “lack of awareness about attractions in Ena city and lack of attractive marketing tools to promote these attractions to foreigners.”

Through the “ideation” stage, the team came up to designing a map paired with an audio guide as an interactive proposal for locals and foreign users. “Map” with “audio guide” concepts addressed feasibility and innovativeness requirements identified earlier. Maps could be placed in areas with high traffic, so users would obtain information about Ena's main attractions. They could also serve as advertisements in addition to being a useful navigation tool. It would provide an authentic feeling of merging with people, culture and technology to achieve the theme of harmony. Thus, the team named the solution as “Harmony Guide.”

Their first prototype presented to the panel members was the map of Ena city paired with two QR-codes that were meant to direct tourists to two versions of an audio-recording. One version of it was made in English, the other one in Japanese. Both of them contained the same information - explanation about the background of “noren” curtain and other Japanese crafts. Each member contributed by making audio-recordings in multiple languages.

After the “iteration” stage, on behalf of Mr Kato, Ms Naruse provided language translation and interpretation to relay comments to improve the prototype via email correspondence and the messenger application. The team created a second version of the “Harmony Guide” prototype after the additional comments. They developed an extended location map that highlighted local restaurants and local inns, more languages and background music with Japanese instruments. At the “testing” phase, Ms Kato, Ms Naruse, and 2 other invited stakeholders formed the evaluation panel to select the “Harmony Guide” system as the most desirable solution out of the five proposals the cohort developed.

**Figure 2:**
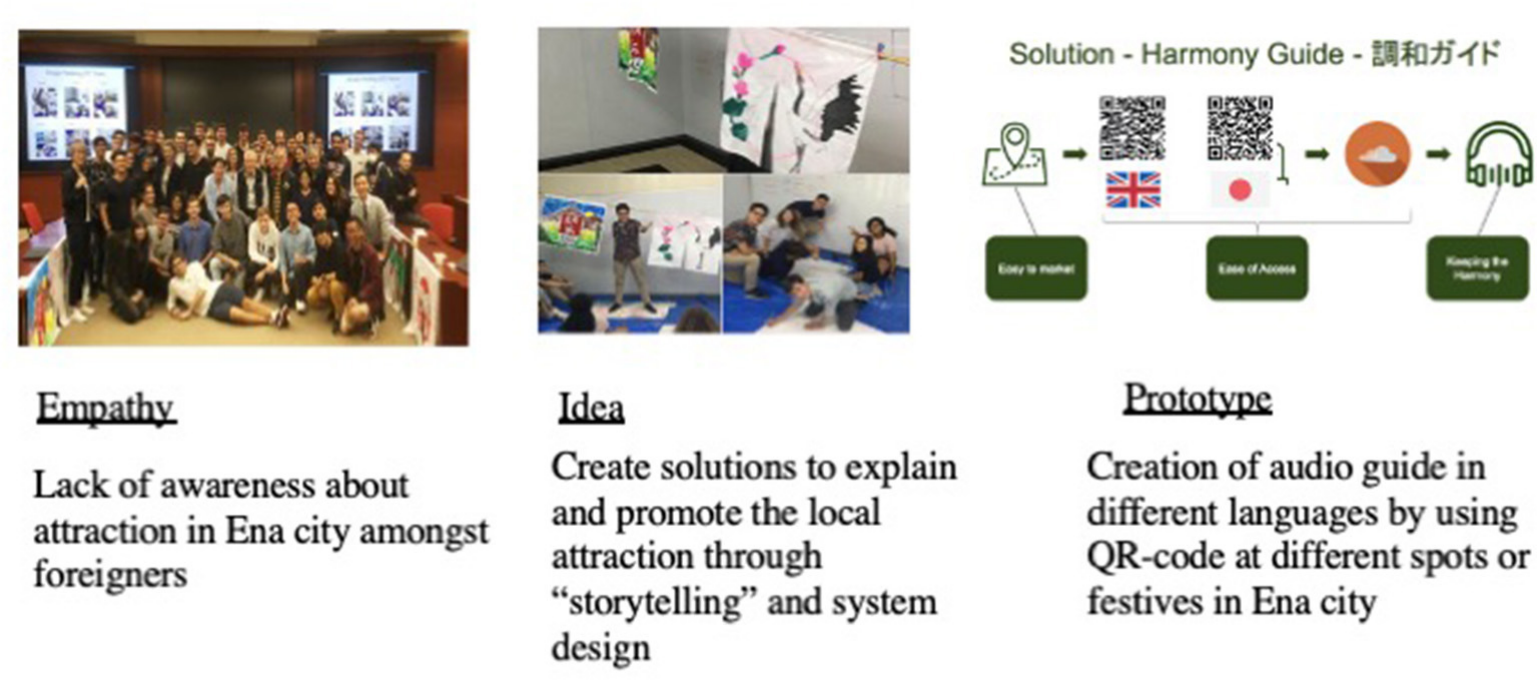
“Harmony Audio” system for Ena estate.

Beyond the project, Mr Kato invited key members in Team B to showcase their prototype and proposal plan at the opening ceremony of the annual “noren” festival held in Ena city. The solution was demonstrated and presented to the city mayor and about 150 residents. The project was written and published into two business case studies registered under the university case centre for academic discussion and research purpose. The prototype and outcomes are summarized in Table 4 and Figure 2.

## Contributions of Citizen Science to Social Innovation

The Kimi No Mori and Ena examples illustrate the manner in which citizen science and social innovation intersect. They also highlight design thinking as a methodology which enables both fields to work together and where the goal of citizen science is the alleviation of a social problem. This section details the contributions that arise when design thinking, citizen science and social innovations are employed.

Employing the design thinking approach in citizen science produces a positive impact on student teams. It enables the creation of innovative solutions, as well as the development of a citizen driven model which could be implemented by the community in the future.

### Induced Creativity of Student Teams and Social Innovation

From the social innovation perspective, the students felt more “motivated and inspired” to deal with real life cases and real people, rather than fictitious characters and narratives from textbooks. The students gained the ability to develop their logics through the two-way interpretation and collaboration with citizens, in order to create the solutions that are desired by the communities.

### Benefits of Design Thinking to Citizen Science

Design thinking is a useful approach because it extends several opportunities for citizen science to occur. Firstly, where science is often associated with sample sizes and large data, design thinking permits experiments to be conducted employing fewer data points. With reference to the two cases, instead of having to identify a large sample of residents, the key appointed citizens (persona and intermediary) may participate in the provision of a new service so as to design the new service. Design thinking permits the scientists or policy makers to explore the options in the development of a new solution to obtain primary data as initial analysis and to consider conducting massive surveys if the solution is deemed feasible.

Secondly, design thinking permits small experiments to be conducted. Experiments of this nature are less costly compared to mounting a full study. Part of the costs are borne by the citizens who volunteer their time and efforts. In contrast, in late scale scientific studies, participants might have to be provided with incentives. In the studies, citizen personas provided the local resources as “raw materials,” such as provision of blueberry jam by Ms Kitakaze and “noren” curtain materials by Mr Kato, in order for students to have physical experience to empathy and induce the latent needs of the citizen within a small scale experiment. The creation of two-way communication via formal emails and informal social media platforms have also enabled the collaboration with minimizing any preliminary fieldwork transportation and hospitality expenses.

Next, the value of these small experiments extends beyond the experimentation, whereby the results of such experimentation is the prototype, the solution. The prototype could be a simulation, a mock-up, a program design, a web page, or a model. The advantage is that the solution is made tangible and visible to the citizens for their iteration and testing. There is proof of concept which has a major significance if adoption of the innovation is intended. In the studies, the proof of concept for both “Digital Blueberry” video and “Harmony Audio” system were ascertained at the “iteration” and “testing” phase for a minimum of two rounds involving the citizen involvements. Social innovation is achieved at the points of proof of concept, while adoption of the two solutions are pending at the end of experiment.

There is the advantage for subsequent citizen scientific endeavours because of the endorsement by the citizens who participated. Their account of their role in the study would encourage the participation of others. The presence of a member from the local community having participated in a project that has potential benefits, will resonate with the rest of the community. There is the promotion of such activities through word of mouth. In the studies, arising from the endorsement of “Harmony Audio” systems by Mr Kato and Ms Naruse, the proposal and prototype were presented to the city mayor and more residents at the “noren” festival opening ceremony. Similarly, the contribution and value of the “Digital Blueberry” video has won the “community film” award in the film festival organized by the university and watched by about 100 student audiences.

Finally, design thinking provides a means to enlist student teams and others who are trained in the methodology as the scientists in citizen science. With citizen science being engaged in social innovation, it would enable the possibility of scaling up efforts with the solutions developed being contextualized to their sites and needs of the citizens, as student teams could be deployed. Furthermore, in harnessing the online solutions such as SKYPE, distance does not hamper any of these efforts.

## Conclusion and Recommendations

From the foregoing discussion, it can be seen that there is merit for citizen science to consider adding design thinking as a methodology where the citizen science activities involve the development of solutions for community needs. The two projects illustrate how design thinking enables two fields of enquiry to operate and produce satisfactory outcomes for the two estates. They make a strong case for design thinking as a means for citizen science to develop social innovations and that citizen science can extend into the realm of social innovation with the citizens involved in citizen science through the design thinking approach.

As to the study itself there is a need to note important limitations and recommendations on how to organize design thinking for future citizen science projects. One limitation is the need to control the influence of cultural differences on the creativity and relevance of solutions for the residents. While it could be argued that the cultural differences mean that there are fresh sets of eyes examining the situation, the countervailing argument would be the lack of empathy that arise because of them. Next, there is a need to consider “post- design thinking” activity to the continuity of the proposal and application of the prototype for sustainability purposes. It is necessary to equip residents with basic skills to continue testing and using the prototype at the local level, with or without the “handholding” by social innovators. Last but not the least is inclusion of appropriate citizen platforms or events, such as town festivals, in order to showcase the prototypes and solutions to the community at large. It would expedite the awareness and even adoption of prototypes by citizens who are ready for social innovation.

Further research is needed to explore the intersection of citizen science and social innovations. There is much that each field can learn from the other to enhance their efforts for the betterment of life for the citizens.

## Data Availability Statement

The datasets presented in this article are not readily available because they contain information that could compromise the privacy of research participants. Requests to access the datasets should be made to goi_hc@gsm.nucba.ac.jp.

## Ethics Statement

Written informed consent was obtained from the individual(s) for the publication of any potentially identifiable images or data included in this article.

## Author Contributions

All authors listed have made a substantial, direct and intellectual contribution to the work, and approved it for publication.

## Conflict of Interest

The authors declare that the research was conducted in the absence of any commercial or financial relationships that could be construed as a potential conflict of interest.

## Footnotes

^1^The features of SKYPE can be found at https://www.skype.com/en/features/.
